# Globular Adiponectin, Acting via AdipoR1/APPL1, Protects H9c2 Cells from Hypoxia/Reoxygenation-Induced Apoptosis

**DOI:** 10.1371/journal.pone.0019143

**Published:** 2011-04-28

**Authors:** Min Park, ByungSoo Youn, Xi-long Zheng, Donghai Wu, Aimin Xu, Gary Sweeney

**Affiliations:** 1 Department of Biology, York University, Toronto, Canada; 2 Institut Pasteur Korea, Seoul, South Korea; 3 AdipoGen Inc., Songdo Technopark, Incheon, South Korea; 4 The Smooth Muscle Research Group, Libin Cardiovascular Institute of Alberta, Department of Biochemistry and Molecular Biology, University of Calgary, Calgary, Canada; 5 Guangzhou Institute of Biomedicine and Health, Guangzhou, China; 6 Department of Pharmacology, Faculty of Medicine, University of Hong Kong, Hong Kong, China; The University of Kansas Medical Center, United States of America

## Abstract

Cardiomyocyte apoptosis is an important remodeling event contributing to heart failure and adiponectin may mediate cardioprotective effects at least in part via attenuating apoptosis. Here we used hypoxia-reoxygenation (H/R) induced apoptosis in H9c2 cells to examine the effect of adiponectin and cellular mechanisms of action. We first used TUNEL labeling in combination with laser scanning cytometry to demonstrate that adiponectin prevented H/R-induced DNA fragmentation. The anti-apoptotic effect of adiponectin was also verified via attenuation of H/R-induced phosphatidylserine exposure using annexin V binding. H/R-induced apoptosis via the mitochondrial-mediated intrinsic pathway of apoptosis as assessed by cytochrome c release into cytosol and caspase-3 activation, both of which were attenuated by adiponectin. Mechanistically, we demonstrated that adiponectin enhanced anti-oxidative potential in these cells which led to attenuation of the increase in intracellular reactive oxygen species (ROS) caused by H/R. To further address the mechanism of adiponctins anti-apoptotic effects we used siRNA to efficiently knockdown adiponectin receptor (AdipoR1) expression and found that this attenuated the protective effects of adiponectin on ROS production and caspase 3 activity. Knockdown of APPL1, an important intracellular binding partner for AdipoR, also significantly reduced the ability of adiponectin to prevent H/R-induced ROS generation and caspase 3 activity. In summary, H/R-induced ROS generation and activation of the intrinsic apoptotic pathway was prevented by adiponectin via AdipoR1/APPL1 signaling and increased anti-oxidant potential.

## Introduction

The increasing prevalence of overweight and obesity and their association with cardiovascular diseases has generated great interest in investigating potential molecular mechanisms linking obesity and cardiovascular disease [1]. Obesity is clearly associated with myocardial structural and functional changes in both humans and animal models [1] and it is widely accepted that obesity will eventually lead to an increased incidence of heart failure. Nevertheless, whereas obesity increases the risk of myocardial infarction (MI), many recent reports now indicate a significant post-MI survival benefit in obese patients [2]. Hence, there is currently a critical requirement to understand the systematic and cellular mechanisms whereby obesity may both elicit MI and yet in some cases protect from subsequent events.

The cardioprotective properties of adiponectin have recently been established [3], [4]. Plasma level of adiponectin is lower in obese individuals and many human studies have suggested hypoadiponectinemia as an independent risk factor for cardiac disorders [3], [5], [6], [7]. Circulating adiponectin occurs as trimeric, hexameric or oligomeric complexes of monomers and cleavage to produce the C-terminal globular domain has also been proposed as an important regulatory step in adiponectin action since this C-terminal fragment can mediate potent physiological effects [8], [9]. The globular and full length forms of adiponectin exhibit different affinities for two adiponectin receptor (AdipoR) isoforms [10] and have been shown to mediate distinct effects [11], [12], [13], [14]. An important role for APPL1 in mediating signaling downstream of AdipoR has recently been characterized such that overexpression or knockdown of APPL1 can result in increased or attenuated adiponectin signaling and effects, respectively [15], [16], [17], [18], [19].

Cardiomyocyte apoptosis is now established as an important remodeling event occurring in end stage cardiomyopathy [20]. Several studies have now demonstrated an anti-apoptotic effect of adiponectin on the heart [21], [22], [23], [24], [25], [26]. However, a major unresolved question is whether the mechanism of action involves AdipoR1 and APPL1. Here we used hypoxia-reoxygenation induced apoptosis in H9c2 cells, an established *in vitro* model for mimicking ischemia/reperfusion of cardiomyocytes [27], to examine the cellular mechanisms responsible for the anti-apoptotic effects of adiponectin.

## Materials and Methods

### Materials

Dulbecco's modified eagle medium (DMEM) was obtained from Gibco Laboratories (Grand Island, NY, USA). Penicillin/streptomycin from Wisent Inc. (Quebec, Canada). The hypoxia chamber was purchased from Billups-Rothenberg, Inc. Mitsubishi Gas Chemical Company, Inc. (Tokyo, Japan) kindly provided the anaerobic pouch (*kenki for cells)* keeping 95%N_2_ and 5% CO_2_ level. We used CM-H2DCFDA from Molecular Probes, Invitrogen, the Caspase 3/CPP32 Colorimetric assay kit from MBL Intl., and Antioxidant capacity assay kit from Sigma Aldrich. Annexin V-FITC Apoptosis Detec.tion Kit I is from BD Biosciences (Canada), the Mitochondrial/Cytosol Fractionation kit is from BioVision (CA, USA). All siRNAs were purchased from Ambion, Inc., and TransIT-TKO reagent was from Mirus Bio Corporation. We used lipofectaimne 2000 from Invitrogen for plasmid transfection. We globular adiponectin from AdipoGen (AG-40A-0006) and produced polyclonal APPL1 antibody in-house. Primary antibodies for AdipoR1/2 were from Phoenix Bio-Tech Corp. (Toronto, Canada); the antibody for cytochrome c was from BD biosciences (Canada). HRP-conjugated anti-rabbit secondary antibody was from Cell Signaling Technology (Beverly, MA). Enhanced chemiluminescence reagent was purchased from PerkinElmer Life Science (Burlington, ON, Canada).

### Cell culture and hypoxia/reoxygenation treatment

Commercially available H9c2 rat embryonic cardiac myoblasts (ATCC) were grown in Dulbecco's modified Eagle medium (DMEM) supplemented with 10% fetal bovine serum (FBS) and 1% (v/v) streptomycin/penicillin (Wisent Inc, Quebec, Canada) at 37°C, 5% CO_2_. When cells reach about 80% of confluence in appropriate culture dishes, cells were pre-starved using DMEM supplemented with 0.5% FBS for 2 hrs and then pretreated with gAd (2.5 ug/ml) for 1 hr. After the pretreatment, simulated ischemia/reperfusion was achieved by culturing the cells in 0.5% FBS DMEM with or without adiponectin in a hypoxia chamber, saturated with 5%CO_2_/95%N_2_ and supplemented with an anerobic pouch (Mitsubishi Gas Chemical Company, Inc.)at 37°C for 21 hrs and following reoxygenation (1–6 h) using 0.5% FBS DMEM with or without adiponectin in the normal incubating condition.

### Detection of DNA fragmentation by TUNEL via laser scanning cytometry

TUNEL (Terminal deoxynucleotidyl transferase-mediated dUTP nick end-labeling) assay was performed using In Situ Cell Death Detection Kit, Fluorescein (Roche Applied Science, Quebec, Canada) according to the manufacturer's protocol. Briefly, cells were grown on cover slips in a 12 well plate and after the proper treatment, cells were fixed in 4% paraformaldehyde and permeabilized using 0.1% Triton X-100 in 0.1% sodium citrate. Then, cells were incubated in TUNEL reaction mixture for 1 hr at 37°C. Coverslips were mounted on microscope glass slide with DAKO mounting medium and analyzed using immunofluorescent confocal microscopy or laser scanning cytometry (CompuCyte Corp), essentially as previously described [28].

### Annexin V binding assay

We used Annexin V Alexa Flour 488 (Molecular Probes, OR) to detect the phosphotidylserine exposure to the outer surface of cell membrane by following the manufacturer's protocol. Briefly, cells were grown on cover slip in a 12 well plate. After the desired treatment, cells were washed with cold PBS and 1x binding buffer (10 mM HEPES pH 7.4, 140 mM NaCl, 2.5 mM CaCl2). Cells were then incubated in Annexin V Alexa Flour 488 (1∶20 dilution) and propidium iodide (1 ug/ml) diluted in 1x binding buffer for 15 min. Then cells were washed twice with 1x binding buffer and the coverslips were mounted on microscope glass slides with Dako florescent mounting medium (DakoCytomation, Missisauga, Canada). Annexin V positive cells were excited and detected at 495/519 nm by a confocal microscopy (Olympus Flouview) and fluorescence quantitated using NIH Image software.

### Detection of cytochrome c release from mitochondria

Cytochrome c release was detected using Western blot analysis after mitochondria/cytosol fractionation. For the fractionation, we used a mitochondria/cytosol extraction kit (BioVision, USA) according to the manufacturer's protocol. Briefly, cells were grown in a 6 cm cell culture dish. After the proper treatment, cells were washed with PBS and resuspended with 100 ul of 1x Cytosol Extraction Buffer Mix containing DTT and protease inhibitors. After 10 min incubation on ice, the cells were scraped off from the dish and collected in a microtubes and syringed 20 times, and then centrifuged at 3000 rpm for 10 min at 4°C. The supernatant were collected and centrifuged at 13,000 rpm for 30 min at 4°C. Then the supernantant was collected as the cytosol fraction, and the pellet was resuspended with 50 ul of the Mitochondria Extraction Buffer Mix containing DTT and protease inhibitors, and it is saved as the mitochondrial fraction. The equal amount of each fraction, 15 µg of cytosol fraction and 10 µg of mitochondrial fraction, were loaded for Western blot analysis using anti-cytochrome c at 1∶500 dilution (BD pharmingen).

### Measurement of caspase 3 activity

The Caspase 3/CPP32 Colorimetric assay kit (MBL, MA) was used to measure the activity of caspase 3 according to the manufacturer's protocol. Cells were grown in a 6 well plate. After the proper treatment, cells were resuspended in 50 ul of chilled lysis buffer on ice for 10 min, and cells were scraped off from the plates and centrifuged at 8,000 rpm for 1 min. The supernatnat was transferred to a microtube, and 50∼100 ug of protein was diluted to 50 ul cell lysis buffer for each assay. Then, 50 ul of 2x Reaction Buffer containing DTT and 5 ul of 4 mM DEVD-pNA substrate were added to each assay and incubated at 37C for 2 hours. After the incubation time, 100 ul of each sample was transferred to each well in a 96 well plate, and read at 405 nm in a microplate reader. Cell lysates were also analyzed by Western blotting with an antibody (Cell Signaling, Beverly, MA) which allows detection of inactive procaspase 3 and activated cleaved caspase 3.

### Western blotting

This was performed as described by us previously [29] using specific antibodies to AdipoR1 at 1∶1000 and APPL1 at 1∶3,000 dilutions. Appropriate horseradish peroxidase-conjugated secondary antibody (anti-rabbit at 1∶10,000 dilution) was used and proteins detected by the chemiluminescence method. Equal loading of protein is routinely ensured by β-actin analysis.

### Measurement of intracellular ROS

We used CM-H2DCFDA (Molecular Probes, Invitrogen) to measure the intracellular ROS during the reoxygenation (1–6 hr) according to the manufacturer's protocol with a few modifications. Briefly, cells were grown in a 6 well plate and after the proper treatment, cells were washed with PBS and incubated in pre-warmed PBS containing the probe in a final working concentration of 5 uM for 30 min at 37°C. Then the PBS solution was removed and the cells were resuspended in microtubes using Trypsin and PBS (Wisent Inc, Quebec, Canada) via centrifugation and pipetting. The cells were resuspended in 500 ul PBS, and 100 ul of the solution was transferred to a 96 well plate for monitoring the fluorescence intensity (485 nm/527 nm) with a microplate reader.

### Antioxidant Capacity Assay

We used commercially available antioxidant assay kit (Sigma-Aldrich) to measure total antioxidant capacity after gAd (2.5 ug/ml, 1 hr) treatment or ascorbic acid (100 uM, 1 hr) as the positive control. In presence of H_2_O_2_, formation of a ferryl myoglobin radical oxidizes the ABTS (2,2′-azino-bis(3-ethylbenzthiazoline-6-sulfonic acid) to its radical cation, which is green in color and can be determined spectrophotometically at 405 nm. Briefly, cells were grown in a 6 well plate and after the proper treatment, cells were washed with PBS and homogenized in 300 ul of cold 1 x assay buffer. The supernatant collected after centrifuge at 12,000 g for 15 min was used for the assay. The antioxidant capacity was quantified by a Trolox standard curve.

### Co-immunoprecipitation after transfection with AdipoR1-Flag plasmid

Cells were grown in normal growth medium in a 6 well plates. Once cells reach 80% of confluency, a plasmid encoding AdipoR1(C)-Flag (3 ug) was transfected in to H9c2 cells using lipofectamine 2000 (Invitrogen). After 18 hours of transfection, the medium was replaced by normal growth medium and then treated with gAd (2.5 ug/ml) after another 24 hours. After the treatment cells were homogenized in lysis buffer containing 1% Triton X-100. Cell homogenate was incubated with a monoclonal anti-Flag antibody (Sigma-Aldrich) and Protein G sepharose (GE healthcare) to pull down the AdipoR1 protein complex for the further analysis by western blot.

### siRNA-mediated knockdown of AdipoR1 and APPL1

Cells were grown in normal growth medium in a 6-well plate. Once cells reach approximately 80% confluence, 21-nucleotide small interfering RNA (siRNA) sequences (Ambion, Inc. Austin, TX) designed to knockdown rat AdipoR1 or APPL1 were tested in H9c2 cells. The sequence of siRNAs ultimately used here for providing optimal efficiency were: AdipoR1, GCUCAUGUUGAGAUUUACtt; and APPL1, GCUUAGUUCUUGUCAUGCAtt. 50 nM of each siRNAs were transfected into H9c2 cells using the TransIT-TKO reagent (Mirus Bio Corporation, Madison WI), precisely according to instructions provided by the manufacturer. After 24 hours of incubation, the medium was replaced by normal growth medium and cells were then treated as described previously after another 24 hours. The efficiency of siRNA-mediated AdipoR and APPL1 knockdown was determined by Western blotting cell lysates prepared 48 hrs after the siRNA transfection.

### Statistical analysis

Data are presented as means ± SEM or as otherwise indicated in legends. Statistical analysis was undertaken using student's *t* test and differences between groups were considered significant when *P*<0.05.

## Results

### Adiponectin pretreatment prevents H/R-induced apoptosis in H9c2 cells assessed by DNA fragmentation and annexin V labeling

**Figure 1:**
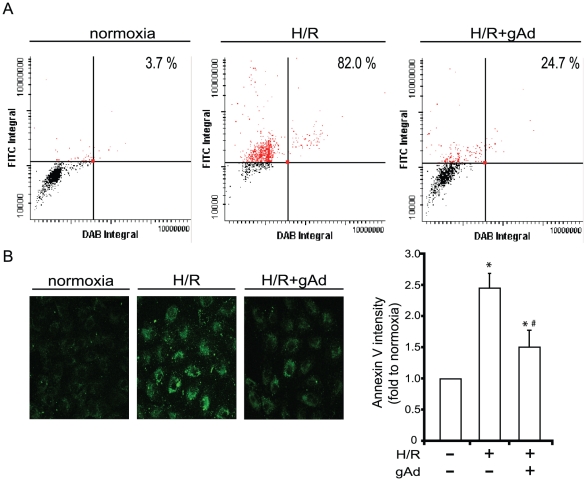
Effect of gAd on H/R-induced DNA fragmentation detected by terminal deoxynucleotidyl transferase dUTP nick end labeling (TUNEL) using laser scanning cytometry and phosphatidylserine externalization detected by Annexin V labeling. Shown in (A) is a representative scatterplot for each condition (normoxia, H/R: 21 h hypoxia followed by 6 h reoxygenation, H/R+gAd (2.5 µg/ml)) where above the horizontal line indicates TUNEL positive cells (red). The quantitative analysis of % TUNEL positive cells is shown inset. Values shown are mean ± SEM from three separate analyses. (B) shows representative immunofluorescent confocal images and the quantitative analysis of intensity of Annexin V positive fluorescence. Values shown are mean ± SEM from n = 3. * indicates p<0.05 with respect to normoxia and # indicates p<0.05 with respect to H/R.

After 21 hours of hypoxia followed by 6 hours reoxygenation we observed a significant increase in DNA fragmentation using terminal deoxynucleotidyl transferase dUTP nick end labeling (TUNEL). This was determined both by laser scanning cytometry (LSC; figure 1A) and by immunoflourescent microscopy (data not shown). The quantitative analysis provided by LSC demonstrated that the H/R treatment resulted in 82±3.2% TUNEL positive cells with only 3.7±0.67% observed under normal conditions (figure 1A). Therefore, H/R induced an ∼22-fold increase in the population of TUNEL positive H9c2 cells (*P*<0.05). There was a statistically significant attenuation of H/R-induced TUNEL positive staining when cells were pretreated with gAd (2.5 µg/ml; 24.7±6.1). In all cases gAd was added 1 hour prior to hypoxia and presence maintained until assays were conducted. We also used annexin-V binding to externalized phsophatidylserine to further investigate apoptotic events under the above conditions. Our data demonstrated a significant increase in phosphatidylserine exposure upon H/R treatment which again was attenuated by gAd pretreatment (figure 1B). The lack of significant propidium iodide staining of cells in these experiments (data not shown) was indicative of apoptotic but not necrotic cell death under H/R conditions.

### Adiponectin attenuates H/R-induced cytochrome c release from mitochondria and caspase-3 activity

**Figure 2:**
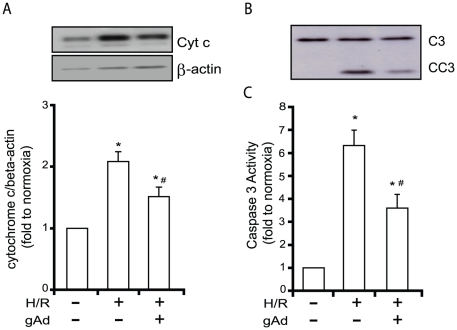
Activation of components of the intrinsic apoptosis pathway upon H/R and their regulation by gAd. (A) shows a representative Western blot of cytochrome c expression in cytosolic fraction with quantitative analysis. (B) A representative western blot showing levels of procaspase 3 (C3) and cleaved caspase 3 (CC3) under each condition with quantitative analysis of caspase 3 activity using DEVD-pNA substrate shown in (C). In all cases values shown are mean ± SEM from n≥3 where * indicates p<0.05 with respect to normoxia and # indicates p<0.05 with respect to H/R.

We prepared cytosolic and mitochondrial fractions from cells exposed to H/R ± adiponectin pretreatment to demonstrate release of cytochrome c from mitochondria after hypoxia followed by 6 hours reoxygenation (figure 2A). Quantitative analysis of immunoblots demonstrated that the level of H/R-induced cytochrome c release to cytosol was significantly attenuated in gAd-treated cells (figure 2A). Correspondingly, there was also a significant increase in caspase 3 activity induced by H/R, determined via analyzing procaspase cleavage (figure 2B) or using a caspase 3 activity colorimetric assay kit (figure 2C). Preatment of cells with adiponectin attenuated caspase 3 activity (figure 2B & 2C).

### Adiponectin attenuates H/R-induced ROS generation via enhancing anti-oxidative potential in cells

**Figure 3:**
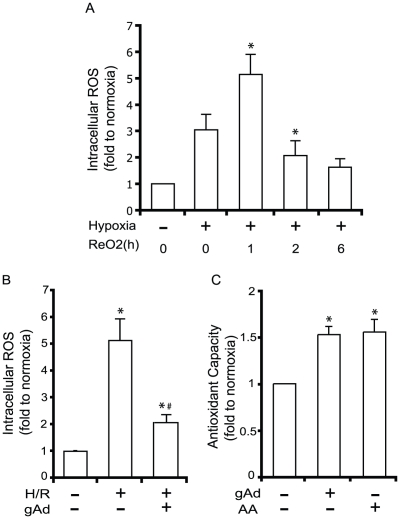
Anti-oxidative potential of gAd against H/R induced ROS production in H9c2 cells. (A) shows intracellular ROS levels after 21 h hypoxia followed by reoxygenation (≤6 hr). Data are expressed as fold relative to normoxia. (B) Effect of gAd (2.5 µg/ml) pretreatment on intracellular ROS after hypoxia and 1 hr reoxygenation (H/R). (C) shows ability of gAd (2.5 µg/ml) to increase the total antioxidative capacity in H9c2 cells. Ascorbic acid (100 µM) was used as a positive control of this assay. In all cases values shown are mean ± SEM from n≥3 where * indicates p<0.05 with respect to normoxia and # indicates p<0.05 with respect to H/R.

Intracellular reactive oxygen species (ROS) play a major role in initiating apoptosis via the intrinsic mitochondrial dependent pathway during ischemia/reperfusion or H/R. Here we demonstrated that hypoxia alone increased the level of intracellular ROS and, furthermore, that 1 hour of reoxygenation induced the highest magnitude of increase in ROS production (figure 3A). After reaching the peak level of ROS, cells restored normal intracellular ROS levels as reoxygenation progressed up to 6 hours (figure 3A). We found that gAd (2.5 µg/ml) pretreatment significantly reduced the peak intracellular ROS level measured after 1 hr reoxygenation following hypoxia (figure 3B). This effect can be explained by the ability of adiponectin to increase the anti-oxidative capacity of these cells (figure 3C).

### AdipoR1 and APPL1 mediate the anti-apoptotic effects of gAd

**Figure 4:**
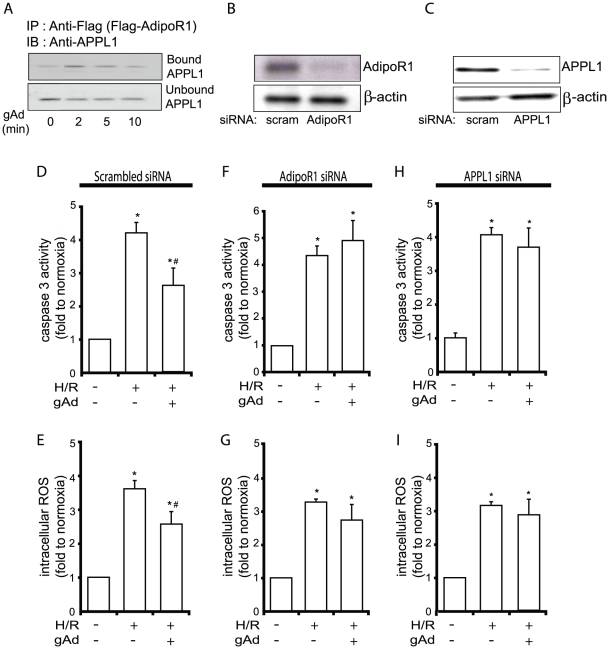
The role of AdipoR1 and APPL1 in mediating the anti-apoptotic effects of gAd. (A) shows a representative Western blot of APPL1 after Co-IP using anti-Flag antibody against AdipoR1(C)-Flag. This demonstrated that gAd (2.5 µg/ml) treatment (0–10 min) induces interaction between AdipoR1 and APPL1 in H9c2 cells. siRNA was used to knockdown expression of AdipoR1 or APPL1 and (B/C) show the representative Western blots demonstrating the efficiency and specificity of the approach. (D–I) show the quantitative analysis of caspase 3 activity measured using the DEVD-pNA substrate assay (D/F/H) and intracellular ROS levels (E/G/I) under each conditon (normoxia, H/R, H/R+gAd (2.5 µg/ml)) in H9c2 cells treated with scrambed siRNA or siRNA specific to AdipoR1 or APPL1. Data presented are mean ± SEM from n≥3. * indicates p<0.05 with respect to normoxia and # indicates p<0.05 with respect to H/R.

**Figure 5:**
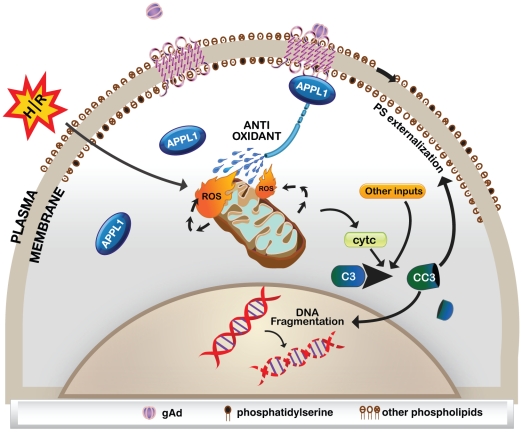
Schematic summary of events. This schematic diagram summarizes the main events which have been established by data in this study. H/R is a stimulus for mitochondrial dependent activation of apoptosis which invloves ROS production and cytochrome c release. This intrinsic pathway of apoptosis involves caspase-3 activation which leads to DNA fragmentation. Another hallmark of apoptosis is the exposure of phosphatidylserine (normally located only on inner membrane) to external surface of plasma membrane. Globular adiponectin, via binding to AdipoR1 and recruiting APPL1 leads to signaling events which enhance anti-oxidant potential in the cells. This is at least in part responsible for reducing ROS production in response to H/R and attenuation of apoptosis.

We demonstrated for the first time in this cell type that gAd stimulated increased binding between AdipoR1 and APPL1, as shown by coimmunoprecipitation study (figure 4A). To then investigate the functional significance of this interaction we used siRNA targeting AdipoR1 or APPL1 to induce loss of function and confirmed the efficiency of knockdown by Western blotting for these proteins. As shown in figure 4B, AdipoR1 expression was significantly decreased by AdipoR1 siRNA but not by scrambled siRNA (figure 4B). We used intracellular ROS production and caspase 3 activity as quantitative assays to determine the functional significance of manipulating AdipoR1 expression. As shown in figures 4D-G, when AdipoR1 siRNA was used the H/R-induced increase in intracellular ROS level and caspase 3 activity remained the same as non-treated cells when treated with gAd, indicating that AdipoR1 knockdown attenuated the protective effect of gAd. We observed that mRNA expression of the AdipoR2 isoform in this cell type was almost 5-fold lower than AdipoR1 and that gAd still prevented H/R-induced ROS production when siRNA was used to knockdown AdipoR2 (data not shown). We also used APPL1 siRNA to examine the role of this AdipoR binding protein in mediating the anti-apoptotic actions of gAd. The efficiency of siRNA to elicit knockdown of this protein was tested by Western blot analysis of cell lysates (figure 4C) and demonstrated significant reduction in APPL1 expression, while scrambled siRNA sequence did not alter endogenous expression (figure 4C). Analysis of the functional significance of APPL1 knockdown showed that the ability of gAd to prevent H/R-induced ROS generation and caspase 3 activity was significantly attenuated by reducing APPL1 expression (figure 4H–I). A schematic diagram showing a summary of these data is shown in figure 5.

## Discussion

The regulation of apoptosis by adiponectin may have widespread implications in obesity linked diseases such as diabetes, cancer, alzheimers and cardiovascular disease [3], [30]. In particular, loss of cardiomyocytes via apoptosis is considered as a contributing factor to progressive deterioration of the hypertrophied left ventricle, ultimately leading to end stage cardiomyopathy [20]. Several recent articles have heightened interest in the effects of adiponectin on cardiomyocyte apoptosis, notably work establishing the cardioprotective effects of adiponectin against apoptosis induced by acute ischemia-reperfusion, or chronic coronary artery ligation models of MI [23], [24]. The former has been shown to occur via AMPK and COX-2 dependent mechanisms [24] and via reduction of oxidative/nitrative stress [25] and activation of Akt by adiponectin was also suggested to play a protective role [22]. Adiponectin also reduced the number of apoptotic cardiomyocytes in mice with viral myocarditis [26]. The existing evidence is convincing and intriguing and provided the impetus for our more detailed mechanistic studies to examine mechanisms of adiponectin action and, in particular, whether adiponectin exerts effects via AdipoR1 and APPL1.

The phenomenon of ischemia-reperfusion induced cell death is important in a variety of circumstances including MI as well as during bypass surgery. Reperfusion of ischemic tissue is vital for long-term survival yet there is also a significant induction of cell death during this phase [31]. Here we used H/R-induced apoptosis in H9c2 cells, often employed previously as an *in vitro* model for ischemia/reperfusion [27]. The contribution of apoptosis and necrosis to cell death of cardiomyocytes during ischemia-reperfusion has been, and remains, controversial [32]. We analyzed annexin-V binding which will recongnize phosphatidylserine exposed on the outer leaflet of the cell membrane as a marker of cell death and in combination with propidium iodide staining of nuclei observed that while a positive annexin-V signal was detected after H/R, PI was excluded suggesting cell death was occurring principally via an apoptotic pathway. Indeed, our further analysis of apoptosis, via DNA fragmentation determined by TUNEL labeling in combination with laser scanning cytometry, demonstrated that H/R-induced a significant increase in TUNEL-positive cells. The anti-apoptotic effects of adiponectin were clearly evident in both of these assays, confirming previous reports of anti-apoptotic effects of adiponectin in cardiomyocytes, and based on these data we then focused more closely on examining the intrinsic apoptotic pathway and mechanisms mediating the effects of adiponectin.

The intrinsic apoptotic signaling pathway has been suggested to be an important component of cardiomyocyte cell death and was recently shown to play an important role in the transition from compensated hypertrophy to heart failure [33]. Pro- and anti-apoptotic Bcl-2 family proteins play an important role in regulating the intrinsic apoptotic pathway and in particular Bax, a principal effector pro-apoptotic Bcl-2 family member plays an important role in H/R induced apoptosis by regulating release of cytochrome c [34]. Generation of reactive oxygen species (ROS) by mitochondria is also an important step in the intrinsic apoptosis pathway stimulated by H/R [35], [36]. In correlation with previous studies, we found that hypoxia alone was a weak stimulus of ROS generation [27], [36] but that reoxygenation rapidly induced a significant increase of intracellular ROS. This is in keeping with previous studies reporting ROS generation during the reperfusion phase of cardiac injury after ischemia [36], [37], [38]. Importantly, we observed a significant decrease of intracellular ROS generation in adiponectin treated cells and that this was likely mediated by the fact that adiponectin directly increased anti-oxidative capacity of these cells. This anti-oxidative effect of adiponectin is supported by previous studies showing that gAd lowered superoxide levels after ischemia/reperfusion in adiponectin knockout mice [25]. Our result can also be correlated to a clinical study which demonstrated that pravastatin increased adiponectin sythesis in the visceral adipose tissue and reduced oxidative stress in men with coronary artery disease [39]. In addition to the anti-oxidative effect of adiponectin, we also found an anti-apoptotic effect of adiponectin in lowering the activity of caspase 3, one of the final executioner proteins in the apoptotic cascade. This work supports previous studies demonstrating an ability of adiponectin to protect not only cardiomyocytes, but also endothelial, neuroblastoma and pancreatic beta cells from caspase-3 mediated cell death [40], [41], [42].

Although many studies have demonstrated the functional role of AdipoR1 in mediating metabolic effects of gAd [10], [43], the role of this receptor in mediating the anti-apoptotic effects of gAd in cardiomyocytes remained to be estabished. Previous work by Kadowaki's group [10] demonstrated that AdipoR1 suppresion reduced gAd binding whereas AdipoR2 suppresion affects fAd binding in C2C12 myotubes since gAd has a higher binding affinity for AdipoR1 and fAd for AdipoR2. We have also demonstrated that metabolic effects of gAd in primary neonatal cardiomyocytes were mediated via AdipoR1 and that AdipoR2 knockdown attenuated fAd actions [29]. In this study, we used siRNAs targeting AdipoR1 to efficiently knockdown expression and identify the functional role. Indeed, AdipoR1 knockdown attenuated the protective effects of gAd on ROS and caspase 3 activity. Our studies described above, together with the small gAd-mediated decrease in H/R-induced intracellular ROS levels are in agreement with the concept of AdipoR1 primarily mediating the effects of gAd. APPL1 (adaptor protein containing PH domain, PTB domain, and leucine zipper motif) has been implicated as an important component of adiponectin signaling by binding to AdipoR and participating in activation of signaling pathways including Akt and AMPK [15], [16], [17], [18], [19]. Overexpression of full-length and PTB domain of APPL1 enhances adiponectin associated signaling events both i*n vitro and in vivo* [18], [19]. However, the physiological relevance of APPL1 in cardioprotection and anti-ischemia induced damage by adiponectin is not clear at the present time. Here, we found that APPL1 knockdown clearly diminished the anti-apoptotic properties of gAd. Our novel observation of an important role for APPL1 in the anti-oxidative/apoptotic effect of gAd correlates well recent studies suggesting that adiponectin protects against myocardial ischemia reperfusion injury via AMPK and Akt dependent signaling mechanisms [22], [24]. A recent study demonstrated that adiponectin can mediate anti-oxidant effects in an AMPK-independent manner [44]. Since both this study and our current data only show partial, albeit statistically significant, AMPK-independent and -dependent mechanisms, respectively, it is likely that both can be involved.

In summary, we document protective effects of adiponectin against H/R-induced apoptosis in H9c2 cells via anti-oxidative effects and attenuation of the intrinsic apoptotic pathway. Of course care must be exercised in extrapolating this data in a cell model to in vivo significance. Nevertheless, documenting that AdipoR1/APPL1 are involved in regulating anti-oxidative effects and subsequently the intrinsic apoptosis pathway is important to establish. Indeed, targeting this mechanism may represent a desirable approach to treat heart failure and, together with future studies, may be of value in encouraging improved therapeutic approaches.
